# Mom Knows Best: The Universality of Maternal Microbial Transmission

**DOI:** 10.1371/journal.pbio.1001631

**Published:** 2013-08-20

**Authors:** Lisa J. Funkhouser, Seth R. Bordenstein

**Affiliations:** 1 Department of Biological Sciences, Vanderbilt University, Nashville, Tennessee, United States of America; 2 Department of Pathology, Microbiology, and Immunology, Vanderbilt University, Nashville, Tennessee, United States of America

## Summary

The sterile womb paradigm is an enduring premise in biology that human infants are born sterile. Recent studies suggest that infants incorporate an initial microbiome before birth and receive copious supplementation of maternal microbes through birth and breastfeeding. Moreover, evidence for microbial maternal transmission is increasingly widespread across animals. This collective knowledge compels a paradigm shift—one in which maternal transmission of microbes advances from a taxonomically specialized phenomenon to a universal one in animals. It also engenders fresh views on the assembly of the microbiome, its role in animal evolution, and applications to human health and disease.

## Introduction

While the human microbiota comprises only 1–3% of an individual's total body mass, this small percentage represents over 100 trillion microbial cells, outnumbering human cells 10 to 1 and adding over 8 million genes to our set of 22,000 [1],[2]. This complexity establishes a network of interactions between the host genome and microbiome spanning gut development [3], digestion [4],[5], immune cell development [6]–[8], dental health [9],[10], and resistance to pathogens [11],[12]. Recent studies have also provided a greater understanding of how the composition of an individual's microbiota changes throughout development, especially during the first year of life [3],[13]. While the general dogma is that the placental barrier keeps infants sterile throughout pregnancy, increasing evidence suggests that an infant's initial inoculum can be provided by its mother before birth [14]–[18] and is supplemented by maternal microbes through the birthing [19] and breastfeeding [20],[21] processes.

While maternal transmission of microbes in humans has attracted considerable attention in the last few years, nearly a century's worth of research is available for vertical transmission of symbionts in invertebrates [22]. Similar to gut bacteria in humans that assist nutrient intake, many insect-associated bacteria function as nutritional symbionts that supplement the nutrient-poor diet of their host with essential vitamins or amino acids [23],[24]. Since these indispensable symbionts cannot live outside of host cells, they cannot be acquired from the environment and are faithfully transferred from mother to offspring [22],[25]. Maternal transmission in invertebrates has been reviewed elsewhere [22],[26],[27], and Box 1 and Box 2 highlight examples of heritable symbioses across invertebrate phyla.

**Box 1. Examples of Maternal Transmission in Marine Invertebrates**

**Marine Sponges (Phylum Porifera)**

Sponges are ancient metazoans that evolved over 600 million years ago as one of the first multicellular animals [83]. In marine sponges, a remarkably large consortium of extracellular microbial symbionts thrives within the sponge's mesohyl, a gelatinous connective tissue located between the external and internal cell layers. Many of these bacterial residents are found in diverse species of sponges with nonoverlapping distributions but not in the surrounding seawater [84]–[86]. These “sponge-specific” microbes are hypothesized to have originated from ancient colonization events before the diversification of marine sponges and are maintained as symbionts through vertical transmission [87]. Independent studies have estimated that up to 33 phylogenetically distinct microbial clusters spanning ten bacterial phyla and one archaeal phylum are vertically transmitted in sponges [41],[84],[86],[88]. Both transmission electron microscopy (TEM) and fluorescent *in situ* hybridization (FISH) studies have confirmed the presence of microorganisms of different shapes and sizes in the oocytes of oviparous sponges [41] and in the embryos of viviparous sponges [43]–[45].

**Vesicomyid Clams (Phylum Mollusca)**

Deep-sea hydrothermal vent communities rely upon chemosynthetic bacteria to harness chemical energy stored in reduced sulfur compounds extruding from the vents. Metazoans that live in this extreme environment harbor chemosynthetic endosymbionts in their tissues that provide most, if not all, of the host's nutrition [89]. Somewhat surprisingly, most invertebrates that live near hydrothermal events acquire their endosymbionts anew from the environment each generation [90],[91], even though chemosynthetic bacteria are crucial for survival in such a harsh habitat. A major exception to this trend is found in the Vesicomyidae family of clams [92]. Vesicomyid clams retain a rudimentary gut and rely primarily on sulfur-oxidizing bacteria sequestered intracellularly within specialized host cells called bacteriocytes in the clam's large, fleshy gills [93]. Vertical transmission via transovarial transmission appears to be the dominant mechanism for maintenance of these thioautotrophic bacterial symbionts given that follicle cells surrounding an oocyte and the oocyte itself are heavily infected with the chemosynthetic bacteria [46],[94].

**Box 2. Examples of Maternal Transmission in Terrestrial Invertebrates**

**Insects (Phylum Arthropoda)**

Insects that thrive on unbalanced diets such as plant sap, blood, or wood depend upon microbial symbionts for the provision of essential amino acids or vitamins lacking in their food source. In turn, hosts provide a wide range of metabolites to their symbionts as well as protection from environmental stressors. This codependence requires faithful transfer of symbionts to all offspring, usually through transovarial transmission [23],[24]. Reproductive parasites, such as the obligate, intracellular bacteria *Wolbachia*, are also widespread in insects and hijack maternal transmission routes to ensure their spread within an insect population (reviewed in [95],[96]).

Pea Aphid (*Acrythosiphon pisum*)

The pea aphid *Acrythosiphon pisum* (Figure 2A) and its nutritional endosymbiont *Buchnera aphidicola* are a preeminent example of obligate mutualism in insects. The ancestral *Buchnera* gammaproteobacteria was acquired by aphids between 160 and 280 million years ago [97] and has since diverged in parallel with its aphid hosts through strict vertical transmission [26],[97]. *Buchnera* are housed within the cytoplasm of bacteriocytes arranged into dual bacteriome structures located in the aphid body cavity adjacent to the ovaries [98], allowing efficient transfer of *Buchnera* symbionts to developing oocytes or embryos during the sexual and asexual phases of aphid reproduction, respectively. At the cellular level, symbiont transfer occurs when maternal bacteriocytes release *Buchnera* symbionts through exocytosis into the extracellular space between the bacteriocyte and oocyte or embryo, which then actively endocytoses the extracellular *Buchnera* symbionts [51].

Cockroaches (Order Blattodea)

Just as insects are morphologically diverse, the mechanisms by which insects transport symbionts to oocytes are highly varied. In cockroaches, *Blattabacterium*-filled bacteriocyte cells migrate from the abdominal fat body to the distantly located ovarioles where they adhere to the oocyte membrane [99],[100]. Interestingly, the bacteriocytes remain associated with the oocyte for eight to nine days before finally expelling their symbionts through exocytosis. The *Blattabacterium* cells then squeeze between the follicle cells surrounding the oocyte and are engulfed into the oocyte cytoplasm via endocytosis just prior to ovulation [100].

Whiteflies (Family Aleyrodidae)

The whitefly circumvents exocytosis of its intracellular nutritional symbiont, *Portiera aleyrodidarum*, by depositing entire bacteriocytes into its eggs. These maternal bacteriocytes remain intact yet separate from the developing embryo until the embryonic bacteriomes form, at which point the maternal bacteriocytes deteriorate [22].

Tsetse Flies, Bat Flies, and Louse Flies (Superfamily Hippoboscoidea)

Members of the Hippoboscoidea superfamily (Order Diptera) are obligate blood feeders that have developed a unique reproductive strategy termed adenotrophic viviparity that offers a different solution to internal maternal transfer of symbionts. Females of this superfamily develop a single fertilized embryo at a time within their uterus (modified vaginal canal) until it is deposited as a mature third instar larva immediately preceding pupation. During their internal development, the larvae are nourished with milk produced by modified accessory glands that empty into the uterus [101]. The milk primarily consists of protein and lipids [102], but it also serves as a reservoir for maternally transmitted microbial symbionts [103]. For example, the obligate mutualistic symbiont of tsetse flies, *Wigglesworthia glossinidia*, is absent from the female germ line and surrounding reproductive tissues but is found extracellularly in the female milk glands and is first detected in tsetse offspring once milk consumption begins during the first larval stage [103].

Stinkbugs (Superfamily Pentatomoidea)

One of the most common mechanisms of external maternal transmission in insects is that of “egg smearing,” which occurs when a female contaminates the surface of her eggs with symbiont-laden feces during oviposition. Upon hatching, offspring probe or consume the discarded egg shells to acquire the maternal bacteria. This mode of transmission is commonly found in plant-sucking stinkbugs, including the Pentatomidae and Acanthosomatidae families [104]. In the Cynidae family of stinkbugs, along with the Coreidae family of leaf-footed bugs, gut symbionts are transferred maternally via coprophagy, in which offspring consume maternal feces, sometimes directly from the mother's anus [22],[104]. Stinkbugs of the Plataspidae family, on the other hand, have developed a unique mode of transmission via a maternally provided “symbiont capsule” deposited on the underside of the egg mass [56]. These capsules are comprised of bacterial cells dispersed throughout a resin-like matrix surrounded by a brown, cuticle-like envelope that protects the symbionts from environmental stressors such as UV irradiation or dissection [57]. After hatching, plataspid nymphs immediately probe the capsules to ingest the symbionts [56],[59].

European Beewolf (*Philanthus triangulum*)

While nutritional symbionts appear to be the most common type of bacteria transmitted via external maternal transmission in insects, the European beewolf (*Philanthus triangulum*) instead cultivates a symbiotic bacteria that protects offspring against microbial infection during development. Beewolves are solitary digger wasps that deposit their offspring in moist, underground nests, making them susceptible to fungal and bacterial infections [105]. To combat these pathogens, female beewolves cultivate *Streptomyces philanthi* bacteria in specialized glands in their antennae, which they copiously spread on the ceiling of the brood cell before oviposition [106]–[108]. After hatching, the larvae take up the bacterial cells and incorporate them into their cocoon that they build before pupation. When adult beewolves emerge from their cocoon in the summer, female beewolves acquire the maternally provided *Streptomyces* symbiont and house them in the female-specific gland reservoirs along each antenna [108],[109].

By integrating previous studies in invertebrates with recent evidence for maternal microbial transmission in humans and other vertebrates, we contend that maternal provisioning of microbes is a universal phenomenon in the animal kingdom. As a result, a considerable new phase of study in heritable symbiont transmission is underway. Thus, this essay presents current evidence for maternal microbial transmission and provides new insights into its impact on microbiome assembly and evolution, with applications to human health and disease.

## Internal Maternal Transmission

At the turn of the twentieth century, French pediatrician Henry Tissier asserted that human infants develop within a sterile environment and acquire their initial bacterial inoculum while traveling through the maternal birth canal [28]. More than a century later, the sterile womb hypothesis remains dogma, as any bacterial presence in the uterus is assumed to be dangerous for the infant. Indeed, studies of preterm deliveries have found a strong correlation between intrauterine infections and preterm labor, especially when birth occurs less than 30 weeks into the pregnancy [29],[30]. Since preterm birth is the leading cause of infant mortality worldwide [31], much attention has focused on identifying the bacterial culprits responsible for spontaneous preterm labor. Surprisingly, most of the bacteria detected in intrauterine infections are commonly found in the female vaginal tract [29], and risk of preterm birth is markedly increased in women diagnosed with bacterial vaginosis during pregnancy [32]. Interestingly, the vaginal microbial community varies significantly among American women of different ethnicities (Caucasian, African-American, Asian, or Hispanic), with African-American and Hispanic women more likely to have a microbiota traditionally associated with bacterial vaginosis (predominance of anaerobic bacteria over *Lactobacillus* species) [33] and a higher rate of spontaneous preterm deliveries (reviewed in [34]).

**Figure 1:**
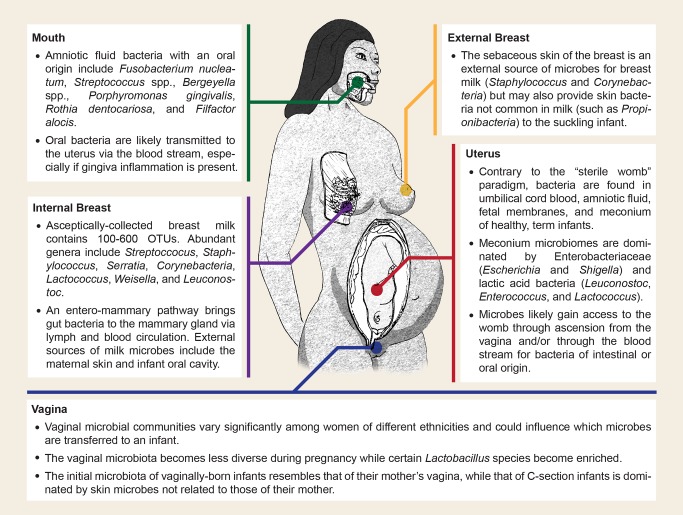
Sources of microbial transmission in humans from mother to child. Cut-away diagram highlighting the various internal and external sources of maternal microbial transmission as well as the species that are commonly associated with transfer from those regions. Regions discussed include the oral cavity [14],[16], the mammary glands [40],[78],[79], the sebaceous skin surrounding the breast [78],[80], the vaginal tract [19],[33],[73], and the intrauterine environment [14],[15],[17],[18],[29],[35]–[37],[40]. Illustration by Robert M. Brucker.

While intrauterine infection and inflammation is important in understanding the etiology of preterm birth, relatively few studies have examined the uterine microbiome of healthy, term pregnancies owing to the sterile womb paradigm. Investigations into the potential for bacterial transmission through the placental barrier have detected bacteria in umbilical cord blood [17], amniotic fluid [14],[35], and fetal membranes [35],[36] from babies without any indication of inflammation (Figure 1). Furthermore, an infant's first postpartum bowel movement of ingested amniotic fluid (meconium) is not sterile as previously assumed, but instead harbors a complex community of microbes, albeit less diverse than that of adults [18],[37]. Interestingly, many of the bacterial genera found in the meconium, including *Enterococcus* and *Escherichia*, are common inhabitants of the gastrointestinal tract [18],[37]. To test whether maternal gut bacteria can be provisioned to fetuses *in utero*, Jiménez *et al.* [18] fed pregnant mice milk inoculated with genetically-labeled *Enterococcus faecium* and then examined the meconium microbes of term offspring after sterile C-section. Remarkably, *E. faecium* with the genetic label was cultured from the meconium of pups from inoculated mothers, but not from pups of control mice fed noninoculated milk. Meconium from the treatment group also had a higher abundance of bacteria than that of the control group. Importantly, the study controlled for potential bacterial contamination from contact between skin and the meconium by sampling an internal portion of the meconium [18]. Thus, this study provides foundational evidence for maternal microbial transmission in mammals.

Other than ascension of vaginal microbes associated with preterm births, the mechanisms by which gut bacteria gain access to the uterine environment are not well understood. One possibility is that bacteria travel to the placenta via the bloodstream after translocation of the gut epithelium. While the intestinal epithelial barrier generally prevents microbial entry into the circulatory system, dendritic cells can actively penetrate the gut epithelium, take up bacteria from the intestinal lumen, and transport the live bacteria throughout the body as they migrate to lymphoid organs [38],[39]. Interestingly, microbial translocation may even increase during pregnancy, as one study showed that pregnant mice were 60% more likely to harbor bacteria in their mesenteric lymph node (presumably brought there by dendritic cells) than nonpregnant mice [40]. Bacterial species normally found in the human oral cavity have also been isolated from amniotic fluid and likely enter the bloodstream during periodontal infections, facilitated by gingiva inflammation [14],[16] (Figure 1).

Overall, the study of internal maternal transmission of microbes in mammals is in its infancy due to the enduring influence of the sterile womb paradigm and to the ethical and technical difficulties of collecting samples from healthy pregnancies before birth. Thus, we still know very little about the number and identity of innocuous microbes that traverse the placenta, whether they persist in the infant, or whether their presence has long-term health consequences for the child. Similarly, we know almost nothing about nonpathogenic viruses or archaea that may be transferred from mother to child alongside their bacterial counterparts. Fortunately, the advent of culture-independent, high-throughput sequencing will serve as a tremendous resource for this field and will hopefully lead to a characterization of the “fetal microbiome” *in utero*.

**Figure 2:**
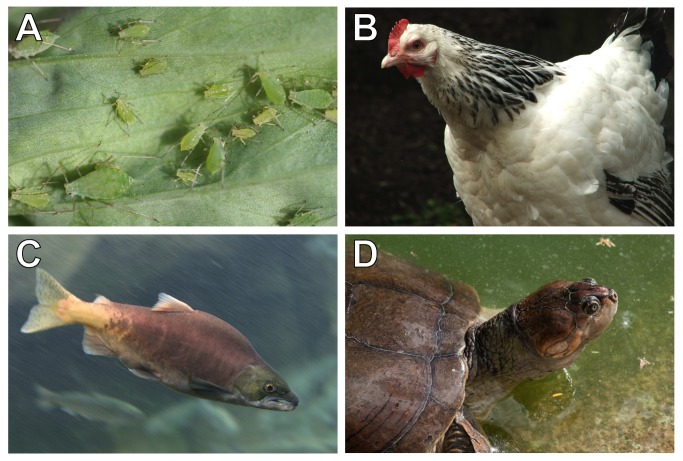
Examples of animals that exhibit microbial maternal transmission. (A) Pea aphid (*Acyrthosiphon pisum*), photo credit: Whitney Cranshaw, Colorado State University/**©**Bugwood.org/CC-BY-3.0-US; (B) Domesticated chicken hen (*Gallus gallus domesticus*), photo credit: Ben Scicluna; (C) Sockeye salmon (*Oncorhynchus nerka*), photo credit: Cacophony; (D) South American river turtle (*Podocnemis expansa*), photo credit: Wilfredor. All photos were obtained from Wikimedia Commons (www.commons.wikimedia.org).

Maternal provisioning of microbes to developing offspring is widespread in animals, with evidence of internal microbial transmission in animal phyla as diverse as Porifera [41]–[45] (Box 1), Mollusca [46]–[49] (Box 1), Arthropoda [50]–[52] (Box 2, Figure 2), and Chordata [19],[53],[54] (Box 3, Figure 2). The presence of maternal transmission at the base of the Animalia kingdom and the surprising plasticity by which microbes gain access to germ cells or embryos in these systems signifies that maternal symbiont transmission is an ancient and evolutionarily advantageous mechanism inherent in animals, including humans. Therefore, we can no longer ignore the fact that exposure to microbes in the womb is likely and may even be a universal part of human pregnancy, serving as the first inoculation of beneficial microbes before birth.

**Box 3. Examples of Maternal Transmission in Vertebrates**

Aside from studies in human and mouse models, very little is known about maternal transmission of microbial communities in vertebrates, especially outside Class Mammalia. Furthermore, research on vertical transmission in nonmammalians has largely focused on maternally transmitted pathogens, especially in animals of agricultural importance like chickens and fish.

**Domesticated Chickens (*Gallus gallus domesticus*)**

Zoonotic *Salmonella* infections acquired from contaminated chicken eggs is estimated to cause more than 100,000 illnesses each year in the United States [110]. In addition to horizontal transmission of *Salmonella* on eggs through surface contamination, direct transovarial transmission also occurs when *Salmonella* colonizes the reproductive tissues of hens (Figure 2B). Depending on the infection location within the female reproductive tract, the bacteria are deposited into the yolk, albumen, eggshell membrane, and/or eggshell of the developing egg before oviposition (reviewed in [111]). Other poultry pathogens, such as *Mycoplasma synoviae* in chickens [112] and *M. gallisepticum*, *M. cloacale*, and *M. anatis* in ducks [113], have also been cultured from the yolk of embryonated eggs, though whether commensal flora are incorporated into the egg is not known.

**Ray-Finned Fish (Class Actinopterygii)**

Several bacterial pathogens of economically important fish are transmitted transovarially in the egg yolk including *Renibacterium salmoninarum*, the agent of bacterial kidney disease in salmonids (Figure 2C), and *Flavobacterium psychrophilum*, which causes bacterial cold water disease in salmonids and rainbow trout fry disease in trout (reviewed in [114]). *F. psychrophilum* has also been found in ovarian fluid and on the surface of eggs of steelhead trout [115]. Additionally, an obligate, intracellular eukaryotic parasite, *Pseudoloma neurophilia*, is a common pathogen found in zebrafish (*Danio rerio*) facilities and has been observed in spores of the ovarian stroma and within developing follicle cells of spawning females, suggesting that it can be vertically transmitted, though it is primarily spread from fish to fish in contaminated water (reviewed in [116]).

**Turtles (Order Chelonii)**

The formation of egg components in the uterine tube and uterus of turtles takes approximately two weeks, providing ample opportunity for maternal transmission of intestinal or reproductive microbes to the egg [117]. One study of unhatched (dead) eggs from loggerhead sea turtle (*Caretta caretta*) nests found several potential pathogens, including *Pseudomonas aeruginosa* and *Serratia marcesans*, in fluid from the interior of the eggs, though environmental contamination of the eggs cannot be ruled out [118]. A similar study of eggs from two species of South American river turtles, *Podocnemis expansa* (Figure 2D) and *P. unifilis*, identified several Enterobacteriaceae species, including *Escherichia coli*, *Shigella flexneri*, and *Salmonella cholerasuis*, in the eggs but not in the environmental samples taken from the turtle nests [119], suggesting that they may have a maternal origin. In support of this hypothesis, a separate study in green turtles (*Chelonia mydas*) that collected eggs directly from the maternal cloacal opening during egg laying isolated *Pseudomonas*, *Salmonella*, *Enterobacter*, and *Citrobacter* from the eggshell, albumen, and yolk. In fact, the yolk was the egg component most heavily infected with bacteria [120]. Altogether, many potentially pathogenic species have been isolated from turtle eggs, but whether these bacteria actually cause disease in turtles or are part of their natural flora remains to be determined.

## External Maternal Transmission

External maternal transmission encompasses any transfer of maternal symbionts to offspring during or after birth. In invertebrates, it is often accomplished by “egg smearing,” in which females coat eggs with microbes as they are deposited [55], or through the provision of a microbe-rich maternal fecal pellet that is consumed by larval offspring upon hatching [56]–[59] (see Box 2). Similarly, human infants are “smeared” with maternal vaginal and fecal microbes as they exit the birth canal [60]–[62] (Figure 1). Several studies have shown that the human neonatal microbiota across all body habitats (skin, oral, nasopharyngeal, and gut) is influenced by their mode of delivery [19],[63]–[65], with infants born vaginally acquiring microbes common in the female vagina while C-section infants display a microbiota more similar to that of human skin [19]. Furthermore, while the microbiota of a vaginally delivered infant clusters with the vaginal bacteria of its mother, the microbiota of C-section babies is no more related to the skin flora of its mother than that of a stranger, indicating that most microbes are transmitted to the neonate from those handling the infant [19]. Importantly, epidemiological data suggest that a Cesarean delivery can have long-term consequences on the health of a child, especially concerning immune-mediated diseases. For example, children born via C-section are significantly more likely to develop allergic rhinitis [66], asthma [66], celiac disease [67], type 1 diabetes [68], and inflammatory bowel disease [69]. These statistics are alarming given that 32.8% of all births in the United States in 2010 were delivered via C-section with similar rates on the rise in most developed countries [70].

The higher rate of immune-mediated diseases in C-section children may indicate that maternally transferred vaginal or fecal microbes are unique in their ability to elicit immune maturation in the neonate. Development of the intestinal mucosa and secondary lymphoid tissues in the gut is contingent upon recognition of microbial components by pattern-recognition receptors on intestinal epithelial cells (reviewed in [71],[72]). It is possible that these receptors cannot properly interact with the community of microbes acquired during Cesarean deliveries, leading to disrupted immune development and an increased risk for immune-mediated disorders in C-section children. Conversely, transmission for thousands of years of vaginal and fecal microbes at birth has likely produced specific human-microbe interactions important for neonatal gut development. In fact, a recent study found that the vaginal microbial community changes during pregnancy, becoming less diverse as the pregnancy progresses [73]; yet, in spite of the general decrease in richness, certain *Lactobacillus* bacterial species are enriched in the vaginal community during pregnancy and are hypothesized to be important for establishing the neonatal upper GI microbiota after vaginal delivery [73].

Breastfeeding provides a secondary route of maternal microbial transmission as shown in humans (reviewed in [74], Figure 1) and nonhuman primates such as rhesus monkeys [75]. In humans, maternal milk microbes are implicated in infant immune system development [76], resistance against infection [77], and protection against the development of allergies and asthma later in childhood [74]. High-throughput sequencing of breast milk from 16 healthy women identified 100–600 species of bacteria in each sample with nine genera present in every sample: *Staphylococcus*, *Streptococcus*, *Serratia*, *Pseudomonas*, *Corynebacterium*, *Ralstonia*, *Propionibacterium*, *Sphingomonas*, and *Bradyrhizobiaceae* [78]. This “core” milk microbiome represented approximately 50% of all bacteria in each sample, with the other half representing individual variation in microbial composition [78]. A similar study found that the bacterial composition in breast milk changes over time: milk produced immediately after labor harbored more lactic acid bacteria along with *Staphylococcus*, *Streptococcus*, and *Lactococcus*, while breast milk after six months of lactation had a significant increase in typical inhabitants of the oral cavity, such as *Veillonella*, *Leptotrichia*, and *Prevotella* [79], perhaps to prime the infant for the switch to solid food. However, as with any DNA-based, culture-independent study that does not discriminate between live and dead bacteria, the number and identity of bacteria detected in these studies should be interpreted with some caution.

Given that milk is only produced temporarily in a woman's life, the origin of milk microbes is still somewhat of a mystery. Breast milk was traditionally thought to be sterile; however, colostrum (the first milk produced after delivery) collected aseptically already harbors hundreds of bacterial species [79]. Breast milk does share many taxa with the microbiota found on sebaceous skin tissue around the nipple [78],[80], and high levels of *Streptococcus* in breast milk may be a result of retrograde flow from an infant's oral cavity back to the milk ducts during suckling [81] since *Streptococcus* is the dominant phylotype in infant saliva [82]. However, the presence of anaerobic gut bacteria in human milk suggests that an entero-mammary route of transfer also exists that may utilize phagocytic dendritic cells to traffic gut microbes to the mammary glands, similar to microbial transfer to amniotic fluid as discussed earlier. To support this hypothesis, Perez *et al.* [40] found identical strains of bacteria in milk cells, blood cells, and fecal samples from lactating women, but more work is needed to directly connect bacterial translocation in the gut to incorporation in breast milk.

Overall, maternal transmission of beneficial microbes in humans has widespread relevance for human health. Evolution with these microbes has resulted in our dependence on them for the proper maturation and development of the immune system and gastrointestinal tract. Somewhat paradoxically, modern medicine designed to prevent infant mortality (such as emergency Cesarean sections and formula feeding) has likely contributed to the rise in immune-mediated diseases in developed countries due to the inherent lack of exposure to maternal microbes associated with these practices. Fortunately, biomedicine is also making strides in finding effective probiotic supplements to promote immune development and ameliorate some of the risks that C-section or formula-fed infants face as children and adults. Hopefully, as we gain understanding of the diversity and function of maternally transmitted microbes in humans, more complete and effective probiotic blends will recapitulate the microbial communities found in vaginally delivered, breast-fed infants and restore the microbe-host interactions that humans depend upon for proper development.

## Conclusions

Since the early twentieth century, the study of maternal microbial transmission has focused heavily on animal systems in which maternal transmission maintains sophisticated partnerships with one or two microbial species. However, with the development of high-throughput sequencing technologies, it is now possible to identify entire microbiomes that are transferred from mother to offspring in systems not traditionally considered to exhibit maternal transmission, such as humans. By expanding the definition of maternal transmission to include all internal and external microbial transfers from mother to offspring, we contend that maternal transmission is universal in the animal kingdom and is used to provision offspring with important microbes at birth, rather than leave their acquisition to chance.

Finally, with microbes contributing 99% of all unique genetic information present in the human body, maternal microbial transmission should be viewed as an additional and important mechanism of genetic and functional change in human evolution. Similar to deleterious mutations in our genetic code, disruption of maternal microbial acquisition during infancy could “mutate” the composition of the microbial community, leading to improper and detrimental host-microbe interactions during development. Maternal transmission is also a key factor in shaping the structure of the microbiome in animal species over evolutionary time, since microbes that promote host fitness, especially in females, will simultaneously increase their odds of being transferred to the next generation. Thus, whether internal or external, the universality and implications of maternal microbial transmission are nothing short of a paradigm shift for the basic and biomedical life sciences.

## Abbreviations

FISH: fluorescent *in situ* hybridization
GI: gastrointestinal tract
TEM: transmission electron microscopy
